# Bacteriological and physico-chemical assessment of wastewater in different region of Tunisia: impact on human health

**DOI:** 10.1186/1756-0500-4-144

**Published:** 2011-05-22

**Authors:** Imen Ben Salem, Imen Ouardani, Mouna Hassine, Mahjoub Aouni

**Affiliations:** 1 Laboratoire des Maladies Transmissibles et Substances Biologiquement Actives - Université de Monastir, Tunisia

**Keywords:** Enteropathogenic bacteria, *Escherichia coli*, pathotype, microbiological quality, reclaimed wastewater

## Abstract

**Background:**

In many parts of the world, health problems and diseases have often been caused by discharging untreated or inadequately treated wastewater. In this study, we aimed to control physico-chemical parameters in wastewater samples. Also, microbiological analyses were done to reveal *Salmonella *strains and each *Escherichia coli *(*E.coli*) pathotype.

**Findings:**

Sixty wastewater samples were collected from fifteen different regions of Tunisia. All physico-chemical parameters (pH, residual free chlorine, total suspended solids, biological oxygen demand, and chemical oxygen demand) were evaluated.

For microbiological analyses, samples were filtered to concentrate bacteria. DNA was extracted by boiling and subjected to polymerase chain reaction (PCR) using different pairs of primers.

The mean pH values recorded for the sampling point were above the WHO pH tolerance limit. The total suspended solids (TSS) concentrations varied between 240 mg/L and 733 mg/L in entrance points and between 13 mg/L and 76 mg/L in exit points. In entrance points, the studied wastewater has an average COD concentration that varied between 795 mg/mL to 1420 mg/mL. Whereas, BOD concentration of the wastewater ranged between 270 mg/L to 610 mg/L. In exit points, COD concentration varied between 59 mg/L and 141 mg/L, whereas BOD concentration ranged from 15 mg/L to 87 mg/L.

The bacteriological control of wastewaters showed that, in entrance points, *Escherichia coli *(*E.coli*)  was detected at the rate of 76.6%. Three *E.coli *pathotypes were found: ETEC (53.3%), EAEC (16.6%) and EIEC (6.6%).

Concerning the ETEC isolated strains, 8 of 16 (50%) have only the heat-labile toxin gene, 5 of 16 (31.2%) present only the heat-stable toxin gene and 3 of 16 (18.7%) of strains possess both heat-labile toxin gene and heat-stable toxin gene. In exist point, the same pathotypes were found but all detected ETEC strains present only the "est" gene.

Concerning *Salmonella *isolated strains; percentages of 66.6% and 20% were found in entrance and exit points respectively.

**Conclusions:**

Wastewaters contain a large amount of pathogenic bacteria that present a real impact on human health. Assessment wastewater treatment stations have to consider in account enterobacterial pathogens as potential pathogens that should be correctly controlled.

## Background

In many developing countries including Tunisia, availability of water has become a critical and urgent problem. In fact, the growing levels of pollution and over-consumption of resources require some sort of solution. Therefore high-quality water sources may be required only for drinking purposes, while the quality of water for other domestic uses can be quite variable. To sum up, the need to conserve water has resulted in an increase in the use of treated sewage effluent, or reclaimed water, for many non-drinking purposes such as irrigation especially in places where numerous recreational resort zones, such as golf courses. In these areas, it is very important to implement water conservation and recycling plans for a more efficient use of water [1]

However, reclaimed water used for irrigation contains parasites, bacteria, and disease-causing viruses. This can create potential health hazards for the exposed human population [2].

Bacteria are among the most common microbial pathogens found in wastewater [3-5]. The practice of unintentional indirect reuse in developing countries is largely responsible for the approximately 4 billion cases of diarrhea daily that cause 2.2 million deaths a year, mainly in children under five years of age [2]. The most known examples are salmonellosis caused by some *Salmonella *spp. Dysentery-like infections have also been found to be caused by some strains of enteropathogenic *E.coli *which can be currently classified into five major categories: enteroaggregative *E.coli *(EAEC), enteroinvasive *E.coli *(EIEC), enterohemorrhagic *E.coli *(EHEC), enteropathogenic *E.coli *(EPEC), and enterotoxigenic *E.coli *(ETEC) [6].

There are several ways in which an individual can acquire disease from wastewater use: direct ingestion of the wastewater or aerosols created during spray irrigation may result in infection. In addition, infection may occur from ingestion of pathogens on contaminated vegetation, oysters or other surfaces. Another potential route of exposure is from the ingestion of ground water that has been contaminated by pathogens in irrigation water [7].

Therefore, it is essential to assess the efficiency of the wastewater treatment and to perform microbiological analyses of the final effluent.

For *Salmonella *spp., the determination of the relatedness of strains within a *Salmonella *serotype is a prerequisite for the identification of the sources of infection and for tracing the routes of *Salmonella *dissemination in outbreaks. Since biochemical analysis did not further differentiate between the bacteria assigned to the same *S. enterica *subspecies, other phenotypic and molecular methods have been used [8,9]. Also, for *E.coli*, traditional O, K, and H serotypes, plasmid profiles, and biotypes in general are unreliable indicators of clonal relationships [10-13]. For these reasons, we tend to optimize a multiplex PCR method to detect the major pathogenic bacteria that can affect human health.

The aim of this study is on physico-chemical indicators of wastewaters and on human enteropathogenic (disease-causing) bacteria (*Salmonella *spp., and *Escherichia coli *pathotypes) detection that may be present in reclaimed water using the multiplex PCR method.

## Materials and methods

### Sampling area and sample collection

**Figure 1:**
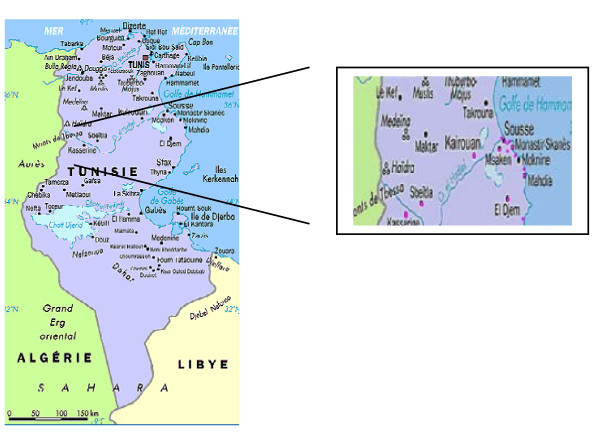
**Tunisian areas involved in the study**.

Wastewater samples were collected, in plastic containers previously cleaned, from fifteen different regions in Tunisia located in the Sahel and the central of Tunisia (Kasserine, Sbeitla, Sousse-Sud, Mahdia, El-Jem, Ksou-essaf, Kairouan, Jammel, Ouardanine, El-frina, Sahline, Dkhila, Msaken, Sousse-Nord, and Sidi-bouzid) (Figure 1). These samples were collected from each site, at the entrance and exit points for each treatment. The duration of treatment was taken into account for samples taken after treatment.

During sampling, sample bottles were labelled and transported to the laboratory. Bacteriological analysis was immediately carried out. Samples were stored in the refrigerator at about 4°C prior for further analysis. It is important to minimize the time between sampling and laboratory analysis to ensure sample integrity: 24 hours (h).

### Determination of physico-chemical indicators

All equipment were checked and calibrated according to the manufacturer's specifications.

#### pH

The pH was determined electrometrically by using the technique recommended in the Standard Methods [14] (GOnDO Electronic Co. Ltd; Taipei; Taiwan).

#### Residual free chlorine

The residual free chlorine content was measured using the N,N-diethyl-p-phenylenediamine (DPD) colorimetric method at the time of sample collection [14].

#### Biological oxygen demand (BOD)

The biological oxygen demand determination of the wastewater samples in mg/L was carried out using standard methods described by Ademoroti [15]. BOD was calculated after the incubation period.

#### Chemical oxygen demand (COD)

Determination of chemical oxygen (COD) demand was carried out using closed reflux method as described by Ademoroti [15].

All of these characteristics measured in entrance and in exit points are summarized in (Table 1).

**Table 1:** Physicochemical characteristics of the wastewater treatment in fifteen different assessment stations in the central of Tunisia

Sample	Colour	Odour	Presence of particules	pH (Mean value)	TSS (Mean value)	BOD (mg/L) (Mean value)	COD (mg/L) (Mean value)	Cl (mg/L) (Mean value)
**Kasserine**								
Enterance point	Colourless	Highly	None	**7.0 ± 1.0**	**617**	**610**	**959**	**437**
Exist point	Colourless	Offensive	None	**8.0 ± 1.0**	**75**	**70**	**120**	**295**

**Sbeitla**								
Enterance point	Colourless	Odourless	None	**7.0 ± 1.0**	**455**	**473**	**941**	**334**
Exist point	Colourless	Odourless	None	**8.0 ± 1.0**	**27**	**27**	**86**	**289**

**Sidi-bouzid**								
Enterance point	Creamy	Not offensive	Suspended	**7.0 ± 1.0**	**520**	**610**	**1130**	-
Exist point	Pink	Not offensive	Suspended	**8.0 ± 1.0**	**76**	**87**	**127**	-

**Mahdia**								
Enterance point	Colourless	Not offensive	None	**7.0 ± 1.0**	**480**	**386**	**1185**	**2760**
Exist point	Colourless	Not offensive	None	**8.0 ± 1.0**	**39**	**39**	**127**	**2690**

**El-Jem**								
Enterance point	Creamy	Not offensive	Suspended	**7.0 ± 1.0**	**336**	**364**	**1010**	**622**
Exist point	Pink	Not offensive	Suspended	**8.0 ± 1.0**	**67**	**83**	**141**	**506**

**Ksou-essaf**								
Enterance point	Colourless	Not offensive	None	**7.0 ± 1.0**	**733**	**411**	**1420**	**719**
Exist point	Colourless	Not offensive	None	**8.0 ± 1.0**	**28**	**25**	**97**	**648**

**Kairouan**								
Enterance point	Colourless	Odourless	None	**7.0 ± 1.0**	**440**	**557**	**1408**	**497**
Exist point	Colourless	Odourless	None	**8.0 ± 1.0**	**25**	**26**	**84**	**426**

**Jammel**								
Enterance point	Colourless	Not offensive	None	**7.0 ± 1.0**	**240**	**270**	**795**	**604**
Exist point	Colourless	Not offensive	None	**8.0 ± 1.0**	**25**	**30**	**106**	**639**

**Ouardanine**								
Enterance point	Colourless	Not offensive	None	**7.0 ± 1.0**	**386**	**472**	**1131**	**622**
Exist point	Colourless	Not offensive	None	**8.0 ± 1.0**	**28**	**31**	**80**	**426**

**El-frina**								
Enterance point	Colourless	Not offensive	None	**7.0 ± 1.0**	**405**	**345**	**1150**	**1163**
Exist point	Colourless	Not offensive	None	**8.0 ± 1.0**	**25**	**30**	**97**	**1246**

**Dkhila**								
Enterance point	Colourless	Not offensive	None	**7.0 ± 1.0**	**348**	**335**	**1040**	**1597**
Exist point	Colourless	Not offensive	None	**8.0 ± 1.0**	**26**	**23**	**71**	**1216**

**Sahline**								
Enterance point	Colourless	Not offensive	None	**7.0 ± 1.0**	**322**	**369**	**1268**	**587**
Exist point	Colourless	Not offensive	None	**8.0 ± 1.0**	**13**	**21**	**70**	**489**

**Msaken**								
Enteance point	Colourless	Not offensive	None	**7.0 ± 1.0**	**604**	**488**	**1085**	**542**
Exist point	Colourless	Not offensive	None	**8.0 ± 1.0**	**24**	**22**	**59**	**486**

**Sousse-Nord**								
Enterance point	Colourless	Not offensive	None	**7.0 ± 1.0**	**338**	**336**	**825**	**1030**
Exist point	Colourless	Not offensive	None	**8.0 ± 1.0**	**33**	**15**	**78**	**728**

**Sousse-Sud**								
Enterance point	Colourless	Not offensive	None	**7.0 ± 1.0**	**409**	**406**	**800**	**710**
Exist point	Colourless	Not offensive	None	**8.0 ± 1.0**	**43**	**48**	**117**	**639**

### Microbiological analysis

For microbiological analyses, two samples each with 1-L volume were collected at each exacting location (Entrance and exit points). Each sample was conducted as follows:

**1) ***Escherichia coli*: 100 mL of each sample was filtered through a 0.45 μm cellulose membrane filter (HA, Millipore, USA) that was placed on 5 mL of Luria-Bertani broth (1% tryptone, 0.5% yeast extract, 0.5%NaCl) and incubated at 37°C for 18/24 h.

**2) ***Salmonella *strains: 100 mL of each sample was filtered through a 0.45 μm cellulose membrane filter. Placed on peptone water, the cellulose membrane was incubated at 37°C for 18/24 h.

Monitoring survival/persistence relies presently on standard methods that may lack sensitivity. These methods are often based on membrane filter techniques and phenotypic identification through culture into selective medium. These methods suffer from limitations imposed by the lack of specificity, antagonistic organism interference, and poor detection of slow-growing or no-cultivable (but viable) organisms [16]. For these reasons we tend to adopt the polymerase chain reaction (PCR) techniques [17,18].

### Molecular bacteriological analysis

Volumes of 100 mL wastewater collected from each entrance and exit points were concentrated by filtration through 0.45 mm filters. The analyses were performed in duplicate - one filter processed for wastewater analysis without dilution and the other for the same sample of water but with dilution factor (1/10) to decrease water charges that can inhibit polymerase chain reaction.

Filters were placed into plastic tubes containing 5 mL of Luria-Bertani broth, followed by overnight incubation at 37°C. After incubation, suspensions were centrifuged for 5 min at 5000 tours/min. The deposit suspension was dissolved into 300 μl of sterile distilled water, vortexed for 5 s than incubated for 20 min at 95°C. The reaction was stopped with abrupt freeze of the suspension (5 min to -20°C). After a second centrifugation, the supernatants were harvested and subjected to polymerase chain reaction using different pairs of primer targeting the genes described below (Table 2).

**Table 2:** Primer sets used to detect enteric bacteria

Bacteria	Primer	Sequence	Amplicon size (bp)	Reference
***Salmonell***	**Hin**	**1750-L **5'- CTAGTGCAAATTGTGACCGCA-3'	236	**Judy et al. 1993 **[25]
***a***		**1751-R **5'- CCCCATCGCGCTACTGGTATC-3'		

	**Hli**	**1788-L **5'- AGCCTCGGCTACTGGTCTTG- 3'	173	**Judy et al. 1993 **[25]
		**1789-R **5'- CCGCAGCAAGAGTCACCTCA3'		

***Escherich ia coli***	**est**	**AL65 **5'-TTAATAGCACCCGGTACAAGCAGG-3'	147	**Hornes et al. 1991 **[21]
		**AL125 **5'CCTGACTCTTCAAAAGAGAAAATTAC-3'		

	**elt**	**LTL **5'-TCTCTATGTGCATACGGAGC-3'	322	**Tamanai-Shacoori**
		**LTR **5'-CCATACTGATTGCCGCAAT-3'		**et al. 1994 **[22]

	**Stx**	**VTcom-u **5'gACCgAAATAATTTATATgTg3'	518	**Yamasaki et al. 1996 **[20]
		**VTcom-d **5'TgATgATggCAATTCAgTAT3'		

	**eae**	**eae1 **5'CTGAACGGCGATTACGCGAA 3'	917	**Gunzburg et al. 1995 **[19]
		**eae2 **5'CCAGACGATACGATCCAG3'		

	**bfpA**	**BF1 **5'AATggTgCTTgCgCTTgCTgC3'	326	**Gunzburg et al. 1995 **[19]
		**BF2 **5'gCCgCTTTATCCAACCTggTA3'		

	**ipaH**	**ipaIII **5'gTTCCTTgACCgCCTTTCCgATACCgTC3'	619	**Sethabutr et al. 1993 **[23]
		**ipaIV **5'gCCggTCAgCCACCCTCTgAgAgTAC3'		

	**aggR**	**aggRks1 **5'gTATACACAAAAgAAggAAgC3'	254	**Ratchtrachenchai et al**.
		**aggRkas2 **5'ACAgAATCgTCAgCATCAgC3'		**1997 **[24]

For *E.coli *detection, The DNA templates were subjected to three multiplex PCRs with specific primers for the detection of the following virulence markers: bfpA (BFP1, BFP2) (structural gene for the bundle-forming pilus of EPEC [19], eae (eae1, eae2)(attaching and effacing lesions of EPEC [19], shiga toxins and their variants (VTcom-u, VTcom-d) of EHEC [20], elt (LTL, LTR) and/or est (AL65, AL125) (enterotoxins of ETEC) [21,22], ipaH (ipaIII, ipaIV) (invasion-associted locus of the invasion plasmid found in EIEC [23] and aggR (aggRks1, aggRkas2) (transcriptional activator of AAF I and AAF II of EAEC [24]. The sequences of primers selected for use in the amplification method matched the sequences of the corresponding genes of ETEC, EPEC, EHEC, EIEC and EAEC.

The minimum criteria for the determination of diarrheagenic *E.coli *were defined as follows: the presence of elt and/or est for ETEC strains, the presence of shiga toxins genes and their variants (stx) for EHEC strains, the presence of bfpA and eae for typical EPEC strains whereas the only presence of eae gene confirms the detection of atypical EPEC strains, the presence of ipaH for EIEC and the presence of aggR for EAEC.

**(i) **Multiplex PCR assay 1 utilizes three primer pairs and detects the presence of shiga toxins and its variants (VTcom-u, VTcom-d), eae, and ipaH genes, generating amplification products of 518, 917, and 619 bp, respectively. **(ii) **Multiplex PCR 2 uses two primer pairs and detects the presence of bfpA and aggR genes, generating amplification products of 326 and 254 bp, respectively. Finally, **(iii) **Multiplex PCR 3 implicated the detection of est and elt genes, generating amplification products of 147 and 322 bp respectively use two primer pairs. Combining molecular results of all of these primers permit an easier detection of the five categories of diarrheagenic *E.coli*.

Whereas, for *Salmonella *detection only one multiplex PCR was done using Hin and Hli primers designed by Judy et al., [25]. These primers were involved in the control of phase variation of *Salmonella *spp. and are only present in *Salmonella *strains. Hin, and Hli primers amplified a 236-pb, and 173-pb fragment respectively.

For each pathogenic bacteria, PCR was performed in 34 μL of reaction mixture containing 5 μl of template DNA, 0.2 mM dNTPs, 2 mM MgCl2, 50 ng of each primer, 5.0 units of Ampli Taq GoldTM polymerase (a Hot Start enzyme from Perkin-Elmer; Applied Biosystems, Canada) and deionised water to make up the volume. Uses of this enzyme resolve problems of inhibitory effect of wastewater components.

For *E. coli *detection, amplification was performed using one cycle at 95°C for 7 min, followed by 35 cycles of 40 sec at 94°C, 40 sec at 50°C and 40 sec at 72°C. Whereas, for *Salmonella *strains we have used the following conditions: Enzyme activation at 94°C for 5 min, and then an additional 33 cycles with heat denaturation at 94°C for 1 min, primer annealing at 63°C for 1 min and DNA extension at 72°C for 1 min. After the last cycle, samples were maintained at 94°C for 10 min to complete synthesis of all strands.

In each experiment, negative buffer (mixture buffer without DNA) and positive (DNA from each reference strain of *E.coli *and *Salmonella *spp. strains) controls were included (Table 3).

**Table 3:** International reference of *E.coli *and *Salmonella *strains used as control for PCRs amplifications

Strain	Internatinal Designation	Positive gene(s)
ETEC	**H10407**	elt

ETEC	**Jep5683**	est

*E.coli *strain	**HB 101**	No virulence gene (negative control)

EHEC	**EDL933 (O157:H7)**	stx and ehxA genes

EPEC	**EPEC2348/69 (O127:H6)**	eae and bfpA

EIEC	**EIEC 11741**	ipaH

EAEC17-2	**EAEC 17-2**	astA and aaf-I genes

*Salmonella Typhimurium*	**ATCC 14028**	-

## Results and discussion

Wastewater can be used for irrigation, but, as reported in other study, it has been generally acknowledged that the greatest hazard associated with the recycling of wastewaters is **(i) **the potential presence of microbial pathogens, that constitutes a risk for the transfer of infections to humans or animals if they are exposed to pathogens in the wastewater, and **(ii) **the chemical component discharged from sewage and industries that contribute to oxygen demand and lead to a destabilized aquatic ecosystem [26,27].

The exposure routes judged to be of main importance were **(i) **direct exposure and accidental ingestion of wastewater, and **(ii) **exposure for aerosol. The contamination of food by water containing known toxin producing organisms can also cause outbreaks of food poisoning [28].

The physico-chemical properties of the wastewater samples collected from different Tunisian regions listed above (as in entrance points or in exit points) are shown in (Table 1). From these results, the levels of pH varied between 7.0 ± 1.0 in the entrance points of each station and 8.0 ± 1.0 for the exit points. Generally exit points show the highest concentration. The mean pH values recorded for all sampling points were above the WHO pH tolerance limit of between 6.00 and 9.00 for wastewater to be discharged into sea or environment [29]. But, pH values ranging from 3 to 10.5 could favor both indicator and pathogenic microorganism growth [30]. Thus, indicated pH levels seem to support bacterial growth.

The total suspended solids (TSS) concentrations varied between 240 mg/L and 733 mg/L in entrance points and between 13 mg/L and 76 mg/L in exit points (Table 1). Literature classified wastewater TSS as follows: TSS less than 100 mg/L as weak, TSS greater than 100 mg/L but less than 220 mg/L as medium and TSS greater than 220 mg/L as strong wastewater. Results of this study show that in entrance points, wastewater can be classified as strong and so cannot be discharged into sea or used for any task. Whereas, in exit points, all TSS values were less than 100 mg/L which reflects the efficiency of wastewater treatment.

An indication of the organic oxygen demand content of wastewater can be obtained by measuring the amount of oxygen required for its stabilization either as BOD and COD. Biological Oxygen demand (BOD) is the measure of the oxygen required by microorganisms whilst breaking down organic matter. While, chemical Oxygen Demand (COD) is the measure of amount of oxygen required by both potassium dichromate and concentrated sulphuric acid to breakdown both organic and inorganic matters.

BOD and COD concentrations of the wastewater were measured in entrance and exit points of each station.

In entrance point, COD value was important in unit process design in most studied points; In fact, the wastewater has an average COD concentration that varied between 795 mg/L and 1420 mg/L. Whereas, BOD concentration of the wastewater obtained for entrance points ranged between 270 mg/L and 610 mg/L. The concentrations of BOD and COD in entrance sampling points were, for the most part, higher than the WHO values of 50 mg/L and 1000 mg/L for the discharged of wastewater into sea [29]. High BOD and COD concentration observed in the wastewater might be due to the use of chemicals, which are organic or inorganic caused by the inflow of domestic, livestock and industrial waste that contains elevated levels of organic pollutants [31] especially if we know that the most important chemical industries were found in the central of Tunisia.

Interestingly, we have noted that, in exit points, COD concentration varied between 59 mg/L and 141 mg/L, whereas BOD concentration ranged from 15 mg/L to 87 mg/L. These values ​​were lower than those obtained at entrance points which reflect the efficiency of wastewater treatment.

Finally, all studied physico-chemical parameters seem to be less than those found by J.C.Akin [29] in different sampling points in Nigeria.

Physico-chemical parameters such as pH, total suspended solids (TSS), Biological Oxygen demand (BOD) and Chemical Oxygen Demand (COD) have a major influence on bacterial population growth [30]. Also, as wastewaters often have high nutrient loads, high numbers of pathogens can be present, increasing the risk of infections occurring from them.

However, in spite of the fact that most bacterial pathogens can be easily of cultured, there are some difficulties in their identification on isolation media, often requiring the distinction between the pathogenic microorganisms and contaminating saprophytic microorganisms which may also be present in the sample.

Thus, the use of selective media and/or selective isolation methods reduces the number of the target organism recovered but the bacterial strains in the environment enter a state where they are viable but no cultivable (VNBC) [16]. Giving that there are different limitation associated with the established methods used for the detection of the various microbial pathogens in wastewaters, researchers have looked for other more sensitive, accurate and quicker detection methods. One of the most common of the new methods examined involves the use of the polymerase chain reaction (PCR) [32].

In this study we have used the multiplex PCR to detect *Salmonella *strains and to identify the five most frequent *E.coli *pathotypes (Table 4).

**Table 4:** Bacteriological characteristics of the wastewater treatment in fifteen different assessment stations in the central of Tunisia

Sample	Number of analysed stools	MPCR results	
		*E.coli *identification (*E.coli *pathotype) [%]	*Salmonella * identification
**Kasserine**			
Enterance point	**2**	+ (EAEC aggR^+^)	-
		+ (EAEC aggR^+^)	-
Exist point	**2**	+ (EAEC aggR^+^)	-
		+ (EAEC aggR^+^)	-

**Sbeitla**			
Enterance point	**2**	+ (EAEC aggR^+^)	-
		+ (ETEC elt^+ ^, est^+^)	+
Exist point	**2**	+ (EAEC aggR^+^)	-
		+ (ETEC est^+^)	-

**Sidi-bouzid**			
Enterance point	**2**	-	-
		+ (ETEC elt^+^)	+
Exist point	**2**	-	-
		-	-

**Mahdia**			
Enterance point	**2**	+ (ETEC est^+^)	+
		+ (ETEC est^+^)	+
Exist point	**2**	+ (ETEC est^+^)	-
		+ (ETEC est^+^)	-

**El-Jem**			
Enterance point	**2**	+ (ETEC est^+^)	+
		+ (ETEC elt^+^)	+
Exist point	**2**	+ (ETEC est^+ ^)	-
		-	+

**Ksou-essaf**			
Enterance point	**2**	+ (ETEC elt^+^)	+
	**2**	+ (ETEC elt^+^) - -	+
Exist point			
		-	-

**Kairouan**			
Enterance point	**2**	+ (EAEC aggR^+^)	-
		+ (EIEC ipaH^+^)	-
Exist point	**2**	+ (EAEC aggR^+^)	-
		+ (EIEC ipaH^+^)	-

**Jammel**			
Enterance point	**2**	+ (ETEC elt^+ ^, est^+^)	+
		+ (EAEC aggR^+^)	+
Exist point	**2**	+ (ETEC est^+ ^)	+
		+ (EAEC aggR^+^)	+

**Ouardanine**			
Enterance point	**2**	+ (ETEC est^+^)	+
		+ (EIEC ipaH^+^)	-
Exist point	**2**	+ (ETEC est^+^)	+
		+ (EIEC ipaH^+^)	-

**El-frina**			
Enterance point	**2**	+ (ETEC elt^+^)	+
		-	-
Exist point	**2**	-	-
		-	-

**Dkhila**			
Enterance point	**2**	+ (ETEC elt^+^)	+
		-	+
Exist point	**2**	-	-
		-	-

**Sahline**			
Enterance point	**2**	-	+
		+ (ETEC elt^+^)	+
Exist point	**2**	-	+
		-	+

**Msaken**			
Enteance point	**2**	+ (ETEC elt^+ ^, est^+^)	-
		+ (ETEC est^+^)	-
Exist point	**2**	+ (ETEC est^+^)	-
		+ (ETEC est^+^)	-

**Sousse-Nord**			
Enterance point	**2**	+ (EAEC aggR^+^)	+
		-	+
Exist point	**2**	-	-
		-	-

**Sousse-Sud**			
Enterance point	**2**	-	+
		-	+
Exist point	**2**	-	-
		-	-

**Total in Enterance point**	**30**	**23 [76.6]**	**20 [66.6]**

**Total in Exit point**	**30**	**15 **50	**6 **[20]

Wastewaters are treated to eliminate pathogenic microorganisms and prevent waterborne transmission. Our research found that, in entrance points, twenty samples were contaminated with *Salmonella *(66.6%), whereas, in exit points a percentage of 20% was found. Therefore, wastewater treatment reduces but does not guarantee the complete elimination of a putative contamination with *Salmonella*. Numerous studies indicate that treated wastewater contain *Salmonella *strains [33].

To more investigate *Salmonella *strains, molecular serotyping method was done using a PCR technique. Molecular serotyping results showed that, in entrance points, three serotypes of *Salmonella *spp. were found: *Typhimurium*, *Enteritidis *and *Montevideo*; whereas, in exit points only the *Typhimurim *serotype was detected **(personal data)**. In 1999 and 2000, in Spain, the most frequent serotypes isolated from wastewater were *S. Enteritidis *and *S. Anatum *[34].

**Figure 2:**
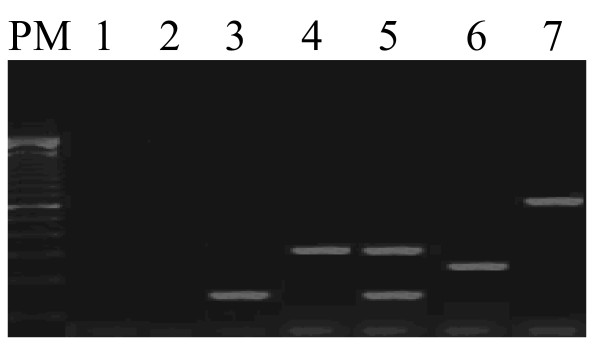
**Agarose gel electrophoresis for combined results of multiplex PCRs (mPCR 1, 2, and 3) amplification of laboratory wastewaters samples.** PM; Lanes: 1, negative control: mixture control; 2, non pathogenic *E.coli *HB101; 3, Sample of Mahdia (ETEC est+); 4, Sample of El-Jem (ETEC elt+); 5, Sample of Jammel (ETEC est+ and elt+); 6, Sample of Kasserine (EAEC aggR+); 7, Sample of Ouardanine (EIEC ipaH+).

Concerning *E.coli *isolated strains, our results should that in entrance points; *E.coli *was detected at the rate of 76.6% (23 samples of 30 samples). This pathogenic *E.coli *belongs to three different pathotypes: ETEC, EAEC and EIEC. ETEC represents the most frequent pathotype with 53.3% (16 samples) then EAEC with 16.6% (5 samples) and EIEC with 6.6% (2 samples) (Figure 2).

Concerning the ETEC isolated strains, 8 of 16 (50%) have only the heat-labile toxin gene, 5 of 16 (31.2%) present only the heat-stable toxin gene and 3 of 16 (18.7%) of strains possess both heat-labile toxin gene and heat-stable toxin gene.

In exit points, ETEC still the most isolated pathotype (53.3%) but we interestingly note that all ETEC strains have only the heat-stable toxin gene. This can be explained by the relative stability of this pathotype in the environment.

As LT-ETEC is thought to be less likely to cause disease than ST or ST and LT ETEC [35], knowledge of the distribution of ETEC toxin phenotypic subgroups may be useful to assess endemic disease incidence. In fact, we have found a high percentage of ETEC strains harbouring "est" gene isolated from Tunisian diarrheal children (42.3%). This pathotype was considered as a pathogen strongly associated with diarrhea that should be taken as a public health problem (data not published).Our environmental findings could explain the important prevalence of ETEC strains harbouring "est" gene isolated from patients.

Moreover, 5 EAEC strains (33.3%) and two EIEC strains (13.3%) were also isolated.

In exit points, water undergoes a decrease in organic contents, and the concentration of pathogens is reduced by antagonistic microorganisms. In fact, the percentage of pathogenic *E.coli *isolated from wastewater was 50% which means that wastewater treatment did not remove all pathogens but gave reduction of 26.6%. The coexistence and competition among strains, species and even different genera can allow interchanges of genetic material, and favor the selection of strains that resist to antibiosis.

Also, the decreased number of bacteria pathogens after wastewater treatment can be explained by adsorption to or incorporation into the secondary sludge, soil or an aquifer. The movement and survival of microorganisms in soil and the subsurface is a highly complex issue which depends on the pathogen type, soil type and condition, water characteristics, the composition and viability of the indigenous microbial population. Numerous other reviews cover the movement of microorganisms particles in surface water, ground water, soil and subsurface soil [36,37].

Disease-causing microbes (pathogens) in these wastewaters can cause diarrhea, cramps, nausea, headaches, or other symptoms. These pathogens may pose a special health risk for infants, young children, and people with severely compromised immune systems [38]. The results of this study should invite us to implement management guidelines for pathogenic bacteria that can affect human health. In particular assessment stations of wastewater treatment have to take in account enterobacterial pathogens as potential pathogens that should be correctly controlled.

The much lack of knowledge that resulted from the inability to adequately detect the microorganisms can be resoled by the use of the multiplex PCR. This technique should lead to more efficient study of the processes and microbial interactions affecting pathogenic microbe survival in the environment. It is also a rapid and sensitive technique as it is able to detect small amount of target DNA in a samples and give a great reduction in the time required to detect pathogenic microorganisms in wastewater samples. But PCR detection of bacteria has generally only been used as a qualitative presence/absence test. Due to the sensitivity of the method, common PCR detection methods are not capable of distinguishing between viable and non- viable pathogenic miroorganisms. This is principally because DNA is relatively stable in the environment, particulary when encased in the membrane of a dead cell.

Moreover, the Ampli Taq Gold TM polymerase (a Hot Start enzyme from Perkin-Elmer) was found to be quite promising as very consistent results were obtained using this enzyme, in all wastewaters samples. The use of this enzyme may resolve problems of inhibitory effect of wastewater components.

## Conclusions

In Tunisia, guideline and criteria for wastewater reuse in all irrigation purpose was done. The microbiological criteria (max) < 1 intestinal nematode egg/l. It does not concern pathogenic bacteria [39]. Despite the fact a number of epidemiological studies about the incidence of infection due to microbial pathogens in wastewater have concluded that there is little or no grater risk to exposed community due to wastewater reuse when compared to the incidence of disease in general community [40,41]; our study highlighted that wastewaters contain a large amount of pathogenic bacteria that present a real impact in human health. In fact, wastewaters treatment reduced the number of pathogenic *E. coli *and *Salmonella *microorganisms but did not remove all pathogens. We also pointed out the role of ETEC as a pathogen strongly associated with diarrhea in our region which can be, in part, explained by the arbitrary reuse of wastewater. Persistence of these pathogenic bacteria, even after wastewater treatment, constituted a potential risk to cause gastrointestinal disease.

## Competing interests

The authors declare that they have no competing interests.

## Authors' contributions

IBS designed the study, carried out the molecular genetic studies, interpreted the data and compiled the manuscript. IO participated in the sample collection, collected the data, and helped in compiling the manuscript. The two authors drafted the manuscript and contributed equally in this work. MH helped to prepare the manuscript. MO conceived the study, participated in the design and the coordination.

All authors (IBS, IO, MH and MO) have read and have approved the final manuscript.
